# Meta‐analysis of elevational changes in the intensity of trophic interactions: Similarities and dissimilarities with latitudinal patterns

**DOI:** 10.1111/ele.14090

**Published:** 2022-08-11

**Authors:** Elena L. Zvereva, Mikhail V. Kozlov

**Affiliations:** ^1^ Department of Biology University of Turku Turku Finland

**Keywords:** biotic interactions, carnivory, elevational gradient, herbivory, macroecology, meta‐analysis, parasitism, predation, terrestrial ecosystems, thermoregulation strategy

## Abstract

The premise that the intensity of biotic interactions decreases with increasing latitudes and elevations is broadly accepted; however, whether these geographical patterns can be explained within a common theoretical framework remains unclear. Our goal was to identify the general pattern of elevational changes in trophic interactions and to explore the sources of variation among the outcomes of individual studies. Meta‐analysis of 226 effect sizes calculated from 134 publications demonstrated a significant but interaction‐specific decrease in the intensity of herbivory, carnivory and parasitism with increasing elevation. Nevertheless, this decrease was not significant at high latitudes and for interactions involving endothermic organisms, for herbivore outbreaks or for herbivores living within plant tissues. Herbivory similarly declined with increases in latitude and elevation, whereas carnivory showed a fivefold stronger decrease with elevation than with latitude and parasitism increased with latitude but decreased with elevation. Thus, although these gradients share a general pattern and several sources of variation in trophic interaction intensity, we discovered important dissimilarities, indicating that elevational and latitudinal changes in these interactions are partly driven by different factors. We conclude that the scope of the latitudinal biotic interaction hypothesis cannot be extended to incorporate elevational gradients.

## Graphical abstract

Meta‐analysis of 226 effect sizes calculated from 134 publications demonstrated a significant but interaction‐specific decrease in the intensity of herbivory, carnivory and parasitism with increasing elevation. We discovered several previously unknown sources of variation in elevational patterns, and revealed important similarities and dissimilarities between latitudinal and elevational changes in the intensity of trophic interactions. We conclude that elevational and latitudinal changes in these interactions are partly driven by different factors and that the scope of the latitudinal biotic interaction hypothesis cannot be extended to incorporate elevational gradients.

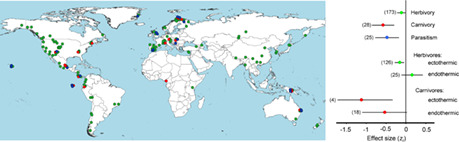

## INTRODUCTION

The exploration of the intensity of biotic interactions in geographical gradients is of both fundamental and applied importance. Studies of these gradients can improve an understanding of how abiotic, biotic and phylogenetic factors concomitantly influence ecosystem structure and functions (Carmona et al., 2020; Chapin III & Körner, 1995) and permit the prediction of temporal changes in ecosystems from contemporary spatial patterns (De Frenne et al., 2013; Tito et al., 2020).

The most important generalisation in this research field is the Latitudinal Biotic Interaction Hypothesis (LBIH), which states that the intensity of biotic interactions reaches its maximum in stable and warm climates and generally decreases from low to high latitudes (Anstett et al., 2016; Schemske et al., 2009; Zvereva & Kozlov, 2021). A similar pattern is frequently observed along elevational gradients (Andrew et al., 2012; Carmona et al., 2020; Hargreaves et al., 2019; Roslin et al., 2017); however, despite this similarity, environmental changes in elevational and latitudinal gradients differ in some aspects. Consequently, whether these two types of geographical gradients in biotic interactions can be explained within a common theoretical framework remains unclear. The further development of a theoretical background for the analysis of environmental gradients in the intensity of biotic interactions is hampered by the great variation in the outcomes of case studies (Andrew et al., 2012; Anstett et al., 2016; Carmona et al., 2020; Moles et al., 2011). Uncovering the sources of this variation may lead to either a wider generalisation (i.e. to addressing phenomena beyond the original domain of LBIH) or to a narrowing of the scope of this hypothesis.

The elevational and latitudinal gradients are both driven by concerted changes in multiple environmental factors (De Frenne et al., 2013; Körner, 2007). However, while temperature decreases with increases in both latitude and elevation, and while changes in plant community structure and productivity follow the same direction, the changes in several other factors differ between these two types of geographical gradients. In particular, day length change considerably with latitude but not with elevation, whereas atmospheric pressure and partial pressure of respiratory gases change with elevation but not with latitude. Finally, UV‐B radiation decreases towards the poles but increases with increasing elevation (Beckmann et al., 2014; Hodkinson, 2005; Körner, 2007; and references therein). Thus, similarities and dissimilarities between elevational and latitudinal gradients in biotic interactions critically depend on the relative importance of these (and many other) environmental factors for the organisms involved in these interactions. Despite the long history of research, this information is still in short supply. We suggest that the comparison of patterns in biotic interactions between elevational and latitudinal gradients would substantially foster an understanding of the mechanisms that shape these patterns.

To allow this comparison, we conducted a meta‐analytical study of elevational changes in trophic interactions paralleling our previous meta‐analysis (Zvereva & Kozlov, 2021) of latitudinal changes in the same interactions. Our ultimate goal was to identify the general pattern in elevational changes in herbivory, carnivory and parasitism and to explore the variations among the outcomes of individual studies, which are associated with characteristics of organisms involved in the interactions and of elevational gradients. We developed testable predictions for the current meta‐analysis based on the conclusions of earlier reviews of elevational studies of herbivory (Andrew et al., 2012; Carmona et al., 2020; Hodkinson, 2005; Moreira et al., 2018; Sundqvist et al., 2013) and parasitism (Péré et al., 2013) and on the regularities found in latitudinal gradients (Zvereva & Kozlov, 2021).

We predicted that (1) the intensity of trophic interactions decreases with increasing elevation. However, the strength or even the direction of elevational changes in these interactions can differ between trophic levels because this type of variation was previously detected in latitudinal gradients: herbivory and carnivory decreased from low to high latitudes, whereas parasitism increased (Zvereva & Kozlov, 2021). For elevational gradients, any particular predictions about variations among trophic levels are hampered by a lack of reviews of elevational changes in carnivory and by inconsistent results of a few primary studies that measured carnivory and herbivory within the same elevational gradients (Bito et al., 2011; Oksanen et al., 1981; Zehnder et al., 2010). Therefore, we could only anticipate that (2) the strength of elevational changes differs between herbivory, carnivory and parasitism.

Considerable decreases in ambient temperature with increases in both latitude and elevation hint that the thermoregulation strategies of organisms may be important predictors of their responses to both geographical gradient types. In line with the higher sensitivity to changes in temperature regimes observed in ectothermic relative to endothermic animals (Huey et al., 2012), several case studies (Hargreaves et al., 2019; Peco et al., 2014; Roslin et al., 2017; Zvereva et al., 2019) reported stronger latitudinal and elevational changes in interactions involving ectothermic animals than in those involving endothermic animals. The previous meta‐analysis (Zvereva & Kozlov, 2021) confirmed that this is a general pattern for both carnivory and herbivory in latitudinal gradients. Therefore, we predicted that (3) the elevational decrease in the intensity of these interactions would be greater for ectothermic than for endothermic consumers.

The direction and strength of gradual changes in the intensity of trophic interactions may also differ between the functional groups of organisms involved in these interactions. For example, this variation could be associated with the feeding habits of consumers, such as external or internal feeding in herbivores (Price et al., 1987) and parasites (Péré et al., 2013), or with the feeding guilds of herbivores (Andrew et al., 2012; Carmona et al., 2020; Kozlov et al, in press). Presuming that internally feeding organisms are better protected from unfavourable abiotic conditions (Péré et al., 2013; Price et al., 1987), we predicted that (4) elevational changes in herbivory would be stronger for externally feeding invertebrates (defoliators and sap‐feeders) than for invertebrates living and feeding within plant tissues (miners, borers and gallers).

Elevational patterns in herbivory may be different on woody and herbaceous plants (Galmán et al., 2018) and on evergreen and deciduous woody plants (Galmán et al., 2018; Zvereva et al., 2020, 2022). A stronger elevational gradient in herbivory on woody plants than on herbaceous plants has been associated with their functional or life‐history traits, whereas the steeper decrease in herbivory in deciduous compared with evergreen woody plants may be expected due to differences in their resource‐use strategies and associated growth rates and antiherbivore defences (Galmán et al., 2018). In particular, this pattern can be expected due to the ability of deciduous plants to maintain the stable level of their nitrogen content in elevational gradients, whereas the nitrogen content in evergreen plant decreases with an increase in elevation (Bai et al., 2015). Therefore, we predicted that elevational changes in herbivory would be stronger (5) for woody than for herbaceous plants and (6) for deciduous woody plants than for evergreen woody plants.

A few comparative studies published to date have found significant variations in elevational patterns of trophic interactions between individual gradients (Hargreaves et al., 2019; Kozlov et al, in press), but the characteristics of elevational gradients that explain this variation are unclear. In latitudinal studies, variation among individual gradients was related to their geographic locations (in terms of midpoint latitude, climate zone and of crossing/not crossing borders between biomes) and their spans (Anstett et al., 2016; Zvereva & Kozlov, 2021). Therefore, we predicted that the strength of the elevational changes observed in trophic interactions depends on (7) climate zone and/or latitude of gradient location, (8) occurrence of abrupt vegetation change (associated with the tree line) within the gradient and (9) the elevational span of the gradient.

The strength of the correlation between the intensity of trophic interactions and latitude or elevation may also be influenced by the methods used for the interaction assessment (Andrew et al., 2012; Anstett et al., 2016). Meta‐analysis (Zvereva & Kozlov, 2021) revealed that the association between trophic interactions and latitude was generally stronger when herbivory or carnivory were measured on standardised plant or prey than on local plant or prey due to the anti‐predator adaptations of local organisms. We anticipated that (10) this difference would also be observed in elevational gradients because local anti‐predator adaptations may exist in high‐elevation populations of plants (Rasmann et al., 2014; Salgado et al., 2016) and animals (Fox et al., 1994).

**FIGURE 1:**
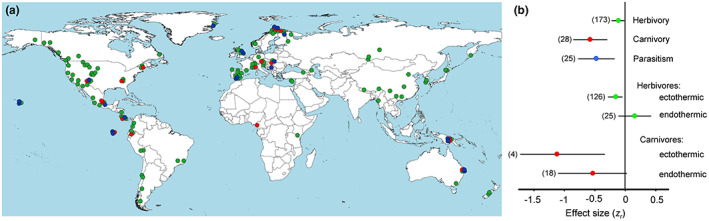
The approximate positions of elevational gradients in herbivory, carnivory and parasitism (a) and strength of elevational changes in the intensity of these interactions—both overall and in relation to the thermoregulation strategies of herbivores and carnivores (b). On the map, dot colours refer to different interactions (consult panel b for explanations); each dot may include several effect sizes (ES) calculated from the same gradient. On the graph, the negative ES indicates a decrease in the interaction intensity with an increase in elevation. Horizontal lines denote 95% confidence intervals; sample sizes (numbers of ES) are shown in parentheses. For statistical analysis, see text.

We have tested these particular predictions by meta‐analysis of the outcomes of 134 publications reporting 226 elevational gradients in herbivory, carnivory and parasitism in multiple mountain regions around the globe (Figure 1a). Based on the results of these tests, we discuss similarities and differences between elevational and latitudinal patterns in trophic interactions to obtain a clearer understanding of whether the patterns observed in these two types of geographical gradients can be generalised within the framework of a common hypothesis.

## MATERIALS AND METHODS

### Search for and processing of studies

We focused our meta‐analysis on trophic interactions in terrestrial ecosystems that are broadly defined as predation (i.e. as consumption of one organism by another organism) and classified into herbivory, carnivory and parasitism, as in previously conducted the meta‐analysis of latitudinal changes in trophic interactions (Zvereva & Kozlov, 2021). We extracted references from earlier reviews of elevational changes in trophic interactions (Andrew et al., 2012; Carmona et al., 2011, 2020; Hodkinson, 2005; Moreira et al., 2018; Péré et al., 2013; Sundqvist et al., 2013), and we then searched for additional publications in the ISI Web of Science using the keywords ‘elevation*’, ‘geographic*’, ‘biotic interactions’, ‘herbivor*’, ‘predat*’, ‘carnivor*’ and ‘parasit*’. The search was completed on 11 January 2022. We did not use unpublished data or grey literature.

To enable comparisons between elevational and latitudinal patterns, we followed the methodology used in our previous meta‐analysis (Zvereva & Kozlov, 2021). We considered studies containing direct quantitative estimates of the intensity of herbivory (the percentage of plant biomass or leaf area lost to herbivores or the proportion of damaged leaves, shoots, flowers, seeds or plants), carnivory (the mortality of prey or the predator attack rates) or parasitism (prevalence; i.e. the percentage of infected hosts). We did not include studies where the interaction intensity was deduced from the abundance of herbivores or predators, because abundance may show variable relationships with the intensity of their impact on plants and on prey (Bito et al., 2011; Tela et al., 2021). We also excluded studies that did not contain data collected from individual elevational gradients but had quantified elevational changes in biotic interactions by combining data obtained in different mountain regions (e.g. Roslin et al., 2017).

We extracted information from studies that fit the following criteria: (1) the data were collected from natural ecosystems, (2) the data were collected from at least two study sites with elevation differences of at least 100 m within the same mountain region and (3) the magnitude of the effect could be calculated from the data or statistics presented in the publication or provided by the authors. From multiyear studies, we extracted the combined result for all years if it was presented in the publication. If the data collected in different years were not combined by the authors, then we selected the year with the highest average value of the character under study. If the study employed some manipulations (e.g. enemy exclusion, insecticide treatment, water treatment), then we selected a control treatment.

### Classificatory variables

Whenever possible, herbivores and carnivores were divided into ectotherms (invertebrates only; we found no data on carnivory by amphibians or reptiles) and endotherms (birds and mammals). All parasites were ectotherms; therefore, they were excluded from this comparison. We classified host plants and prey into natural, permanently inhabiting the study areas, and standardised, that is introduced to all study sites by the researchers (e.g. sunflower seeds or artificial prey).

The herbivory level was considered to be background unless the authors explicitly mentioned an outbreak. Herbivory was divided into folivory (consumption of leaves, sometimes with their supporting branches) and granivory (i.e. seed predation). Folivory was divided into mammalian grazing and invertebrate folivory, whereas granivory was divided into pre‐ and post‐dispersal seed predation. Invertebrate herbivores were divided into exophagous (defoliators and sap‐feeders) and endophagous (miners, gallers, borers) feeders; the latter group also included pre‐dispersal seed feeders.

We divided studies of herbivory into those reporting damage of all plant species in a site or of several dominant species (community‐wide herbivory hereafter) and those reporting damage of a certain plant species or genus (species‐specific herbivory hereafter). Host plants were classified as woody or herbaceous, and woody plants were classified as evergreen (with foliage that remains green and functional through more than one growing season) or deciduous.

Elevational gradients were classified as those located entirely below or above the tree line and those crossing the tree line. We calculated the elevational span of a gradient as the difference in elevation between the highest and lowest sites. The geographical coordinates of study areas (to the nearest degree of latitude and longitude) were extracted from publications or searched on the internet based on information provided in the publication. The attribution of elevational gradients to climate zones (tropical, including subtropics; temperate; and polar, including boreal forests) was based on climate and vegetation at the foot of the mountain and was performed in the same way as in the meta‐analysis of latitudinal patterns (Zvereva & Kozlov, 2021).

### Meta‐analysis

We quantified the strength of the elevational gradients by the *z*‐transformed correlation between elevation and the intensity of the interactions (*z*_*r*_). We estimated the variation in effect sizes (ES) within groups by calculating the heterogeneity index (*Q*_t_). To compare ES among different groups of studies, we calculated the between‐group heterogeneity (*Q*_B_) using a random effects model, and we tested *Q*_B_ against the χ^2^ distribution with the number of groups minus one degree of freedom (Koricheva et al., 2013).

We used three approaches for the ES calculation. When a study reported the data from two or three sites, we calculated Hedge's *d* based on data from the lowest and highest sites. We then converted Hedge's *d* into a Pearson linear correlation coefficient (*r*) following an equation given by Lajeunesse (2013) and calculated the variance of *z*_*r*_ based on sample size (Rosenberg et al., 2013). When the number of sites was four or more, we extracted or calculated the Pearson linear correlation coefficient between the intensity of the trophic interaction and elevation and converted *r* into a *z*_*r*_ value. If the authors provided the *F* statistic, we transformed it into *z*_r_ using a Metawin calculator (Rosenberg et al., 2000). The ES calculated in different ways were of similar magnitudes (*Q*_B_ = 1.09, df = 2, *p* = 0.58), thereby justifying the combination of ES calculated by these methods in our analyses.

We explored the effects of latitude and gradient span on elevational changes in the intensity of trophic interactions by means of a meta‐regression. We searched for publication bias by calculating the Kendall τ correlation between the standardised ES and sample size (Rosenberg et al., 2000); the significant correlation was interpreted as the presence of a small study effect hinting at publication bias (Jennions et al., 2013). Finally, we calculated Rosenthal's fail‐safe number, which shows the number of insignificant studies that are required to turn the significant mean ES into an insignificant one. The fail‐safe numbers exceeding 5*n* (where *n* is the number of studies included in the meta‐analysis) were considered as proof of robustness of the analysis against the insignificant results (Møller & Jennions, 2001).

## RESULTS

### Overview of the data

We discovered 134 publications (dated from 1962 to 2022) that satisfied our criteria, and we calculated 226 ES (Table SM1 in Zvereva & Kozlov, 2022) from these publications. The data were dominated by studies of herbivory (76.5% of ES) on vegetative (leaves, stems) and generative organs (flowers, seeds) of vascular plants. The data on carnivory (12.4%) reflected primarily predation on natural and artificial insect prey and bird nests. The data on parasitism (11.1%) involved mostly parasitoids of insects.

The studies included in our database represented multiple mountain ranges from all continents (excluding Antarctica) and climate zones (Figure 1a). Of 226 ES, 87 were based on contrasts between sites (or groups of sites) located at different elevations, while the remaining 139 were based on correlations with elevation. The number of sites in correlation studies ranked from 4 to 214 (median value: 5 sites). The absolute elevations of individual study sites ranged from −27 to 4640 m above sea level; the elevational span of a gradient varied from 100 to 4640 m (median value: 700 m).

### General patterns

The intensity of trophic interactions generally decreased with increasing elevation (*z*_r_ = −0.21, CI_95_ from −0.30 to −0.12), and this pattern was robust against unpublished studies (the Rosenthal's fail‐safe number equals 6125, i.e. is 27 times greater than the number of studies included in our meta‐analysis). We did not find publication bias in our data (τ = −0.03, *n* = 226, *p* = 0.48).

The overall effect of elevation showed high heterogeneity (*Q*_t_ = 302.5, *df* = 225, *p* = 0.0004), mostly due to different responses of trophic interactions to elevation (Figure 1b; *Q*_B_ = 14.9, *df* = 2, *p* = 0.006). The decreases with increasing elevation were stronger for carnivory and parasitism than for herbivory (Figure 1b); as the result, the elevational decrease in the impact of the third trophic level on their prey (carnivory and parasitism combined: *z*_r_ = −0.53) was more than fourfold greater (*Q*_B_ = 13.9, *df* = 1, *p* = 0.004) than the decrease in herbivory (*z*_r_ = −0.12). The elevational patterns in carnivory and parasitism combined did not show statistically significant heterogeneity (*Q*_t_ = 54.4, *df* = 52, *p* = 0.54), whereas the patterns in herbivory were highly variable (*Q*_t_ = 236.7, *df* = 172, *p* = 0.008).

### Variation related to gradient characteristics

**FIGURE 2:**
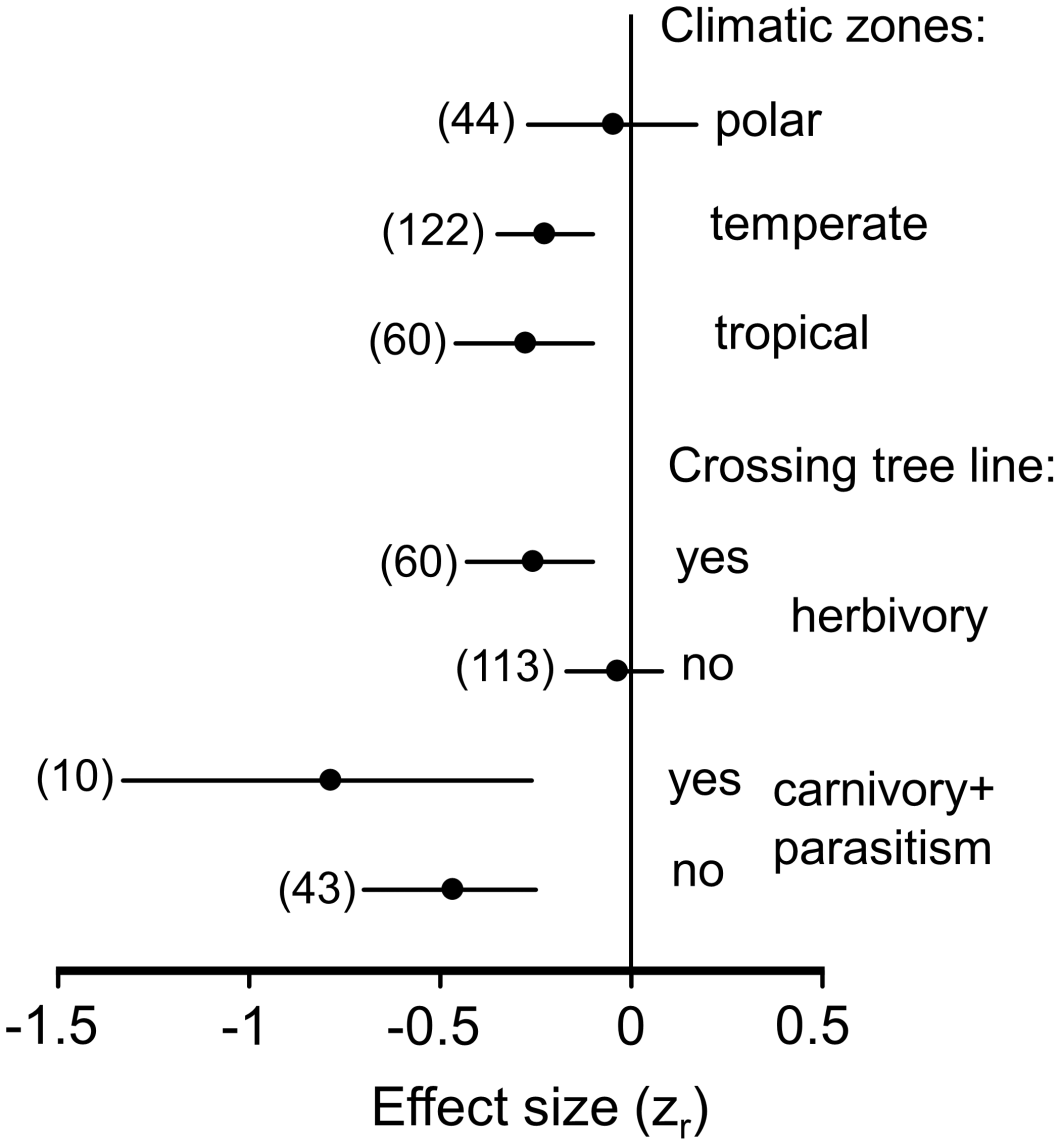
The strength of elevational changes in the intensity of trophic interactions in relation to gradient location in terms of the climate zone (for all interactions combined) and in relation to the tree line (for herbivory and for carnivory combined with parasitism). For other explanations, refer to Figure 1; for statistical analysis, see text.

**FIGURE 3:**
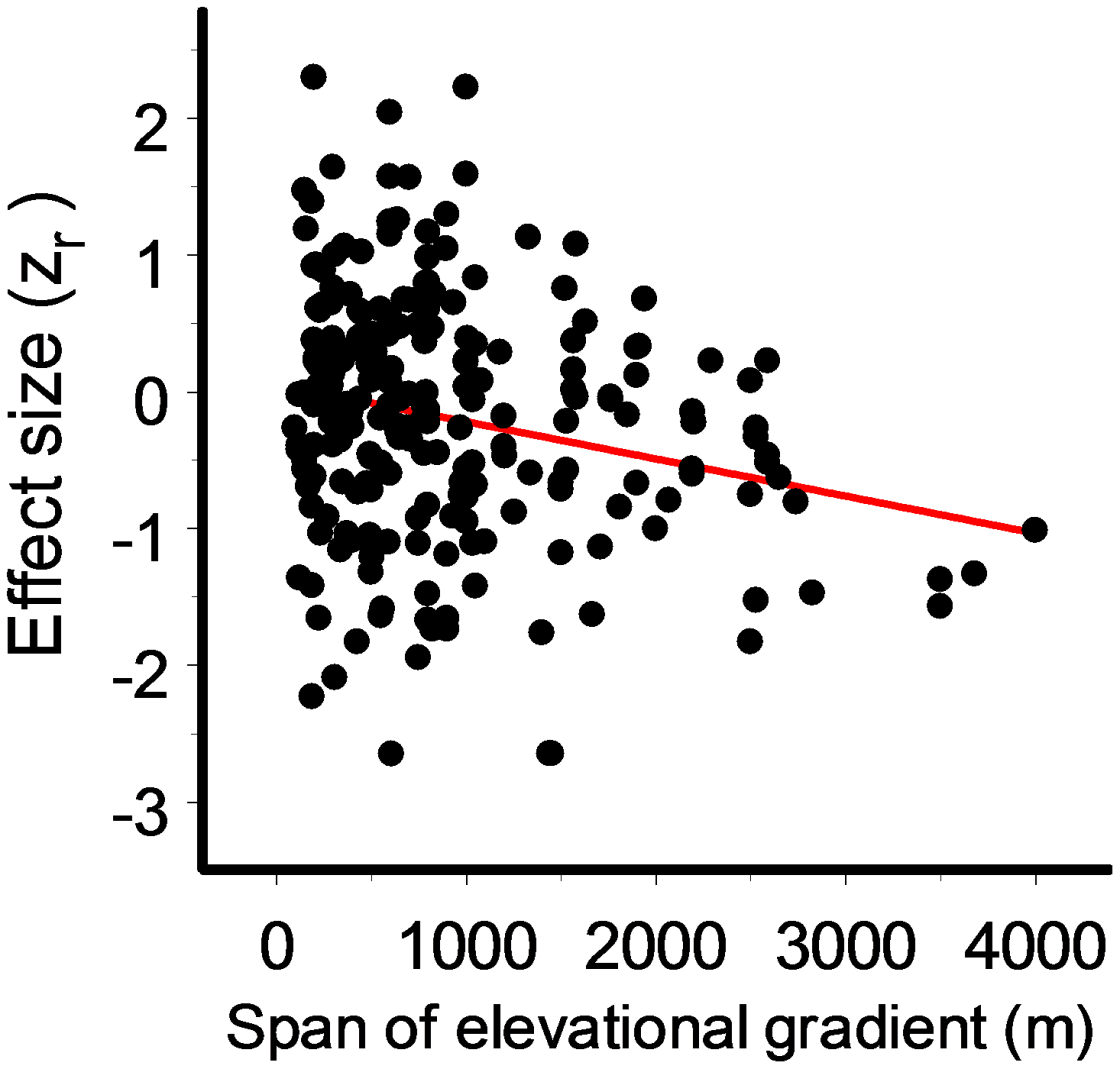
Meta‐regression of the strength of elevational changes in intensity of all trophic interactions to the elevation span of the respective gradients (*Q* = 14.8, *p* = 0.0001).

The association between the intensity of trophic interactions and elevation was stronger in gradients crossing the tree line than in gradients located either below or above the tree line (Figure 2). This difference was valid for all trophic levels and was most strongly expressed in herbivory, which showed significant elevational changes only in gradients crossing the tree line (Figure 2). The strength of the elevational decrease in trophic interactions was threefold greater in gradients located above the tree line than in gradients located below the tree line (*z*_r_ = −0.32 and *z*_r_ = −0.11 respectively), although this difference was not statistically significant (*Q*_B_ = 1.91, *df* = 1, *p* = 0.25). The changes in trophic interactions became stronger with an increase in the elevational span of the individual gradient (Figure 3); this association was significant for gradients that both crossed (*Q* = 9.40, *p* = 0.002) and did not cross (*Q* = 5.84, *p* = 0.016) the tree line.

The elevational decrease in the intensity of trophic interactions was significant in tropical and temperate climate zones, but it was not significant in the polar zone (Figure 2), where no elevational decrease was observed even in gradients that crossed the tree line (*z*_r_ = −0.01). This lack of an elevational decrease was mostly due to herbivory (*z*_r_ = 0. 04), whereas carnivory and parasitism still tended to decrease with elevation (*z*_r_ = −0.38). Meta‐regression of *z*_r_ against latitude was not significant within tropical and temperate zones combined (*Q* = 0.004, *p* = 0.95), whereas *z*_r_ increased in the polar zone (i.e. became less negative) towards the pole (*Q* = 6.51, *p* = 0.01). The latter pattern was mostly due to an elevational gradient in herbivory, which weakened substantially with increasing latitude (*Q* = 16.5, *p* < 0.0001) within the polar zone.

### Variation related to characteristics of organisms involved in interactions

Within both herbivory and carnivory, the elevational decrease was significant only for ectothermic consumers (Figure 1b). Within interactions involving endothermic consumers, the elevational decrease was much stronger for carnivory than for herbivory (Figure 1b; *Q*_B_ = 7.41, *p* = 0.008). Studies using standardised prey or plants yielded a nearly threefold greater magnitude of ES compared to studies that measured carnivory and herbivory on naturally occurring prey or plants (*z*_r_ = −0.39, CI_95_ from −0.65 to −0.14 and *z*_r_ = −0.14, CI_95_ from −0.24 to −0.04 respectively; *Q*_B_ = 3.43, *df* = 1, *p* = 0.09).

**FIGURE 4:**
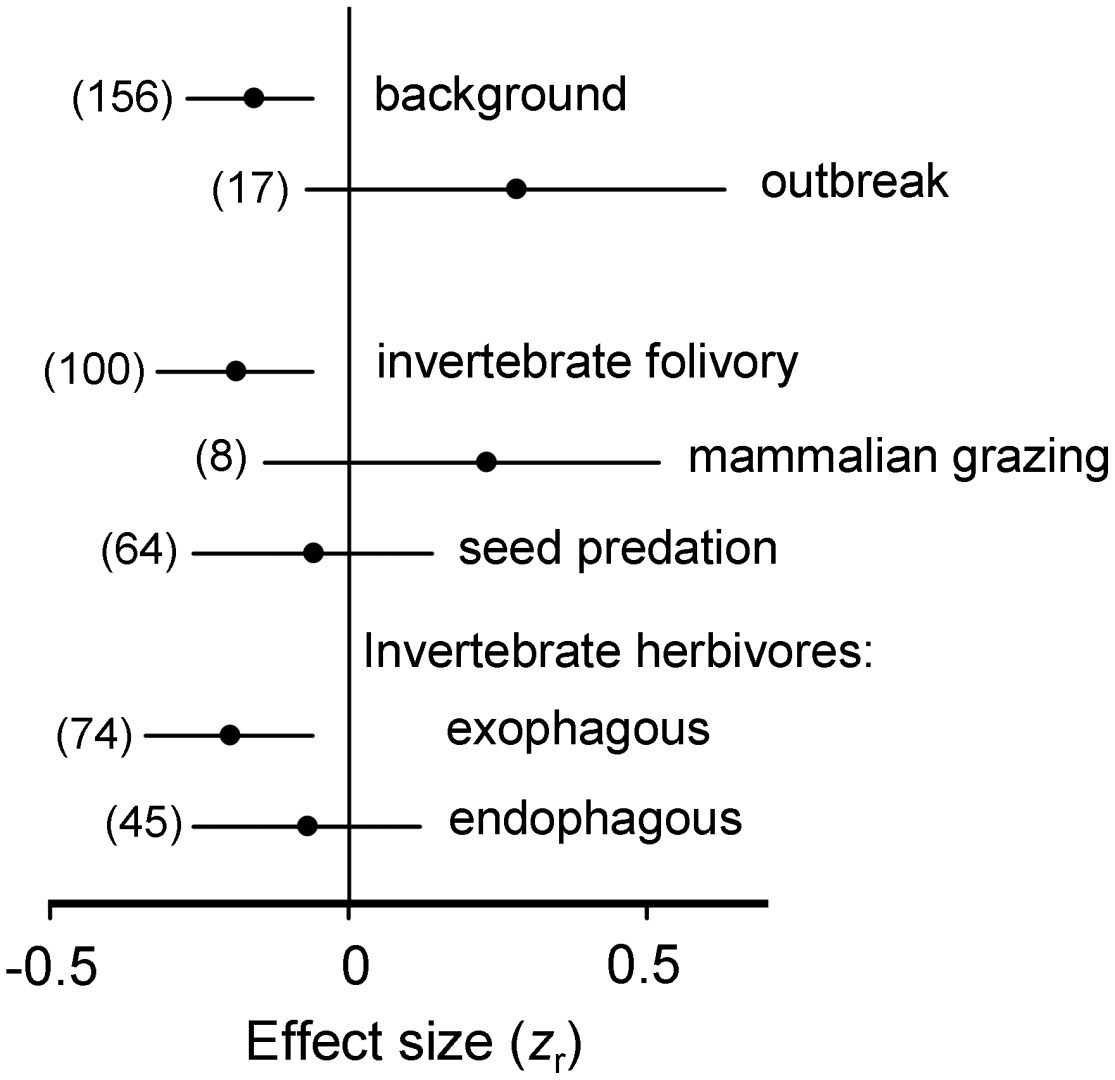
Sources of variations in the elevational changes in the intensity of different kinds of herbivory. For explanations, refer to Figure 1; for statistical analysis, see text.

Invertebrate folivory decreased with elevation, whereas mammalian grazing and seed predation did not show significant elevational changes (Figure 4); pre‐ and post‐dispersal seed predation showed similar patterns (*z*_r_ = −0. 08 and *z*_r_ = −0.04, respectively; *Q*_B_ = 0.03, *df* = 1, *p* = 0.86). Within invertebrate herbivores, the damage imposed by exophagous species (defoliators, sap‐feeders, post‐dispersal seed predators) decreased with increasing elevation, while herbivory imposed by endophagous species (miners, gallers, borers and pre‐dispersal seed predators) did not change with elevation (Figure 4). Across all types of herbivory, background herbivory significantly decreased with increasing elevation, whereas outbreak herbivory tended to increase (Figure 4; Q_B_ = 6.76, *df* = 1, *p* = 0.03).

**FIGURE 5:**
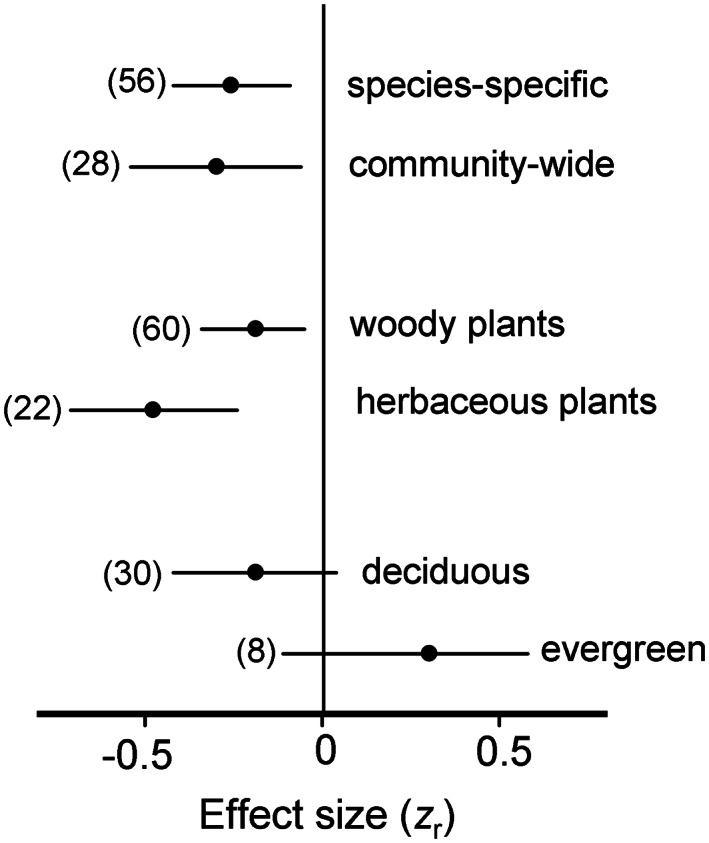
Sources of variations in the elevational changes in the intensity of background invertebrate folivory. For explanations, refer to Figure 1; for statistical analysis, see text.

Within background invertebrate folivory, the elevational changes assessed at the community level were similar to the changes assessed at the species or genus level (Figure 5; *Q*_B_ = 0.10, *df* = 1, *p* = 0.76), and the elevational decrease tended to be greater in herbaceous than in woody plants (Figure 5; *Q*_B_ = 4.38, *df* = 1, *p* = 0.07) due to the tendency of folivory to increase with elevation in evergreen woody species (Figure 5).

## DISCUSSION

### Variation related to the characteristics of organisms involved in interactions

In line with our prediction, we revealed an overall decrease in the intensity of trophic interactions with increasing elevation. However, elevational decreases in carnivory and parasitism were more than fourfold stronger than in herbivory. This result is consistent with an increase in sensitivity to abiotic factors, primarily ambient temperature, with an increase in trophic level (Nelson et al., 2019; Urban et al., 2017; Voigt et al., 2003) and could be explained by the intrinsically greater metabolic rate of top consumers, which are generally more active foragers than herbivores (Urban et al., 2017; Vasseur & McCann, 2005). These differences in temperature sensitivity between trophic levels were previously detected in studies of climate warming effects on trophic interactions (Petchey et al., 1999; Urban et al., 2017; Voigt et al., 2003), but our meta‐analysis shows that these differences also manifest themselves in elevational gradients.

Importantly, the considerable difference in strength elevational decrease of interaction intensity between trophic levels was observed even within interactions involving endothermic organisms, which are less sensitive than ectothermic animals to low temperatures (Huey et al., 2012; Urban et al., 2017). This result indicates that the disproportionate response to elevation among trophic levels is not only due to the different temperature sensitivities of the respective organisms, but it also reflects their different susceptibilities to other factors that change with elevation (e.g. plant community structure and productivity and/or concentrations of respiratory gases), which may similarly affect both ecto‐ and endothermic organisms. Decreases in oxygen concentrations with increasing elevation may be especially detrimental for higher trophic levels, because higher metabolic rates are progressively more constrained by oxygen availability (Rubalcaba et al., 2020). In addition, the model linking relative intensities of herbivory and carnivory with primary productivity (Oksanen et al., 1981) predicts that the role of predation in herbivore regulation should decrease with increasing elevation due to elevational decline in productivity of plant communities.

A high sensitivity to the abiotic environment of high‐elevation regions may result in a sharp decrease in predator density and, consequently, in predation rates. For example ants, which are responsible for 80% of the predation events on insect baits in the Andes, essentially disappear above 1500 m a.s.l. (Camacho & Aviles, 2019). In addition, a positive association between discovery rates of predatory ectotherms and net primary productivity (Kaspari & de Beurs, 2019) could explain the decrease in predation rates with increasing elevation. Similarly, parasitoids in high elevation habitats may suffer from strong winds, which—especially in combination with low temperatures—can reduce their host‐finding success (Vosteen et al., 2020).

The differential sensitivity of trophic levels to changing environment may lead to community destabilisation (Voigt et al., 2003). For example, the stronger elevational decreases in carnivory and parasitism relative to herbivory may disrupt feedbacks that regulate herbivore population dynamics. This disruption is the likely reason underlying one of the most striking results of our meta‐analysis, namely the strong difference observed in the response to elevation between herbivory imposed by animal populations at their background densities versus their outbreak densities.

Herbivore outbreaks are frequently explained by the loss of control from their natural enemies (Mlynarek et al., 2017), particularly in elevational studies (Hoset et al., 2017; Oksanen et al., 1981). Our discovery of stronger elevational decreases in carnivory and parasitism relative to herbivory suggests that, at some elevations, herbivores can be released from regulation by their natural enemies, and this release may create prerequisites for herbivore outbreaks. We suggest that elevational changes in background and outbreak herbivory are driven by different factors: while background herbivory decreases with increasing elevation, mostly due to unfavourable changes in the abiotic environment (Carmona et al., 2020; Hodkinson, 2005; Rasmann et al., 2014), outbreaks may occur at elevated sites due to changes in the biotic environment. More generally, different elevational patterns in background and outbreak herbivory are in line with our earlier prediction (Kozlov & Zvereva, 2017) that abiotic drivers of global change, and temperature in particular, may have different effects on background versus outbreak herbivory.

Among the different types of herbivory, only invertebrate folivory showed significant decreases with increasing elevation, while mammalian grazing and seed predation did not change. This difference likely emerged due to two fundamental sources of variation: the thermoregulatory strategy of animals and their feeding habits. No elevational patterns were observed when herbivory was caused either by endothermic animals (mammalian grazers, vertebrate post‐dispersal seed predators) or by invertebrates feeding within plant tissues (pre‐dispersal seed predators, borers, miners, gallers).

The general lack of an elevational decrease in herbivory caused by internally feeding invertebrates is in line with the hypothesis (Kozlov, Castagneyrol, et al., 2022; Price et al., 1987) that feeding inside plant tissues protects herbivores from the direct impacts of some harmful abiotic factors. This is especially the case for desiccation (Tooker & Giron, 2020), which is one of the major threats faced by insects at high elevations because of increased solar radiation and strong winds (Hodkinson, 2005; Körner, 2007). At the same time, the temperature inside plant tissues follows the ambient air temperature (Levitt, 1980; Price et al., 1987); therefore, the difference in elevational patterns between endophagous and exophagous herbivores indicates that factors other than temperature have contributed to shaping these patterns within invertebrate herbivores.

Plants that differ in functional and life‐history traits may exhibit inconsistent elevational patterns in herbivory (Galmán et al., 2018; Kozlov, Zverev, & Zvereva, 2022; Zvereva et al., 2022). In their global analysis, Galmán et al. (2018) discovered a decrease in leaf herbivory with increasing elevation in woody plants but not in herbaceous plants. By contrast, we revealed a significant decrease in leaf herbivory in both woody and herbaceous plants. This inconsistency may be explained by the different methods used for data analysis: Galmán et al. (2018) combined all data on herbivory from sites located at different mountain ranges, whereas we compared woody and herbaceous plants based on correlations observed within individual elevational gradients. Similar differences between the outcomes of these two types of analysis were previously demonstrated by Kristensen et al. (2020): a decrease in herbivory with elevation was significant in individual gradients, but not significant when data from all gradients were combined. Analyses based on individual gradients may be more efficient in detecting general elevational patterns than analyses combing data from different latitudes, because the latter method does not account for variation between individual gradients. Nevertheless, both Galmán et al. (2018) and our meta‐analysis concluded that elevational changes in herbivory were greater (i.e. more negative) in deciduous than in evergreen woody plants. The latter difference may be partly explained by stronger responses of plant growth and of antiherbivore plant defences (in terms of foliar concentrations of carbon‐based secondary compounds) to temperature in deciduous plants than in evergreen (mostly coniferous) plants (Way & Oren, 2010; Zvereva & Kozlov, 2006).

### Variation related to characteristics of gradients

The decrease in the intensity of trophic interaction becomes stronger with the increase in elevational differences between the lowest and highest sites within a gradient. This is evidently related to greater differences in abiotic conditions and, consequently, to a higher signal (i.e. true elevational differences) to noise (i.e. spatial or temporal variation not related to elevation) ratio. Furthermore, the decrease in the intensity of all studied interactions is stronger in gradients crossing the tree line (i.e. spanning more than one vegetation zone) than in gradients located entirely within a single vegetation zone either below or above the tree line (Figure 2). These results hint at the importance of the indirect impact of elevational changes in abiotic conditions on trophic interactions through vegetation type and other ecosystem properties, which change dramatically across tree lines (Mayor et al., 2017). From a methodological perspective, this result indicates a greater ability of longer gradients to detect elevational changes in trophic interactions. Data on long gradients are in short supply (Figure 3), and we recommend that future studies, whenever possible, use gradients spanning more than 1000 m in elevation.

Only a few studies (Hargreaves et al., 2019; Kozlov, Zverev, & Zvereva, 2022) have explored elevational patterns in trophic interactions across several mountains differing in their geographic position. Based on theoretical considerations, Galmán et al. (2018) suggested that the gradient in herbivory between low and high elevations may be steeper in the tropics than in temperate regions due to latitudinal differences in the magnitude of elevational changes in climatic conditions. However, they found no support for this prediction. Consistently, our meta‐analysis did not reveal any differences in elevational changes between tropical and temperate regions, either for herbivory alone or for all trophic interactions combined.

By contrast, our meta‐analysis revealed that elevational changes in herbivory are small or even absent in high‐latitude mountains, in line with the hypothesis based on a study conducted above the Polar Circle (Zvereva et al., 2022). This phenomenon was also confirmed for all trophic interactions combined, which did not decrease with increasing elevation across boreal and polar localities (Figure 2). Moreover, at high latitudes, the strength of the association between this intensity and elevation weakens with the increasing latitude of the mountain range.

The latitudinal trend in the elevational changes in the intensity of trophic interactions revealed by our meta‐analysis closely corresponds to a nearly constant lapse rate (i.e. change in temperature per unit elevation) between the equator and about 50° N/S, followed by a strong poleward decrease in the lapse rate at higher latitudes (Mokhov & Akperov, 2006; Neumann, 1955). Temperature gradients are seen as a major driver of elevational changes in biotic interactions (Abdala‐Roberts et al., 2016; Andrew et al., 2012); therefore, we suggest that the overall lack of elevational changes in the intensity of trophic interaction at high latitudes is likely explained by smaller lapse rates, consistent with the frequent occurrence of the inverse elevational gradients of temperature in polar mountains (i.e. an increase in temperature with increasing elevation: Pepin et al., 2009; Graae et al., 2012; Kankaanpää et al., 2021). Importantly, a small or even positive lapse rate in high‐latitude mountains, in combination with the substantial decrease in the impact of top consumers, may facilitate herbivore outbreaks, as indicated by a greater fraction of studies reporting outbreak herbivory in elevated sites of this region (39% of all herbivory studies included in our meta‐analysis) relative to temperate and tropical zones combined (8% of studies).

We conclude, that although the strength of the elevational changes in trophic interactions is greater at lower than at higher latitudes, the latitudinal changes in elevational gradients are not gradual. Instead, a sharp shift in the strength of elevational gradients occurs only in boreal and polar regions, while no change is observed between tropical and temperate zones.

### Similarities and differences between elevational and latitudinal changes in trophic interactions

We have revealed multiple similarities between the changes in trophic interactions along elevational and latitudinal gradients, although the magnitude and sometimes even the sign of the effect differ for some subsets of data. Most importantly, we have found a significant overall decrease in the intensity of trophic interactions with increases in both latitude and elevation. However, the strength of this decrease is twofold greater for elevational gradients (*z*_r_ = −0.21: current study) than for latitudinal gradients (*z*_r_ = −0.11: Zvereva & Kozlov, 2021).

The changes in herbivory are similar between latitudinal and elevational gradients (*z*_r_ = −0.15 and *z*_r_ = −0.12 respectively), so the difference in the overall effect outlined above is due to the different responses of the top consumers to environmental changes in these two geographical gradients. Changes in carnivory are almost fourfold stronger with increasing elevation than with increasing latitude (*z*_r_ = −0.58 and *z*_r_ = −0.15 respectively). Even greater differences are observed in parasitism, which significantly increases with increasing latitude (*z*_r_ = 0.18) but significantly decreases with increasing elevation (*z*_r_ = −0.48). Importantly, the elevational decrease in parasitism is consistent with the meta‐analysis by Péré et al. (2013), which only partially overlaps with our meta‐analysis due to the use of different criteria for data inclusion.

We suggest that the greater response of top consumers to elevational gradients relative to herbivores is explained by their greater metabolic demands due to active foraging, as this makes carnivores and parasites especially sensitive to decreased partial pressures of oxygen at high elevations. This hypothesis is in agreement with the lack of differences between herbivory and carnivory in the latitudinal gradient (Zvereva & Kozlov, 2021) because oxygen availability does not change with latitude.

By contrast, the opposite changes in parasitism observed along elevational and latitudinal gradients are difficult to explain. The only abiotic factor that changes in different directions along these two geographical gradients is UV‐B radiation (Beckmann et al., 2014; Hodkinson, 2005; Körner, 2007). Sensitivity to UV‐B radiation increases with the decrease in organism size (Van Atta et al., 2015). Therefore, we suggest that tiny parasitoids are especially vulnerable to increased levels of UV‐B radiation at high elevations. This sensitivity then contributes to their declines at high altitudes but not at high latitudes.

Both latitudinal and elevational changes were significant only for ectothermic herbivores and predators, whereas interactions involving endothermic organisms did not change in either latitudinal or elevational gradients. This similarity, evidently explained by the higher sensitivity of ectotherms to ambient temperatures (Buckley et al., 2012; Huey et al., 2012), confirms that ambient temperature is one of the most important abiotic factors driving both latitudinal and elevational changes in trophic interactions (De Frenne et al., 2013; Peco et al., 2014; Romero et al., 2018).

We found that herbivory and carnivory measured on standardised prey (e.g. non‐native plant species/genotypes or their seeds, artificial models of insects and bird nests) yield a stronger decrease with increases in either latitude or elevation than herbivory and carnivory measured on naturally occurring local prey. This difference can be explained by the evolved local adaptations of native prey or plant in different sites along both gradients, whereas standardised models did not participate in evolution (Freeman et al., 2020). The two meta‐analyses (Zvereva & Kozlov, 2021; and this one) indicate that local anti‐herbivore and anti‐predator adaptations considerably and consistently modify the strength of both latitudinal and elevational gradients in herbivory and predation. Overall, the use of a standard plant or standard prey leads to an overestimation of the strength of the environmental gradients in trophic interactions that actually exist in natural populations. This potential overestimation should be taken into account when interpreting the results of gradient studies.

Both meta‐analyses revealed that geographical patterns in trophic interactions differ sharply between polar zone and the rest of the world. Within polar zone, the latitudinal changes appeared significantly stronger and the elevational changes appeared significantly weaker than the corresponding changes in both temperate and tropical zones (Zvereva & Kozlov, 2021; and this study). These two trends likely have common roots because the low correlation of the intensity of trophic interactions with elevation may be explained by the shallower temperature lapse rate at high latitudes (Mokhov & Akperov, 2006; Sadoti et al., 2018), which is, in turn, related to an increasingly sharp poleward decrease in temperatures in the polar zone relative to the temperate and tropical zones (Terborgh, 1973; Wang & Dillon, 2014). Both meta‐analyses hint at a high specificity of the geographical gradients in the intensity of trophic interactions at high latitudes, and they stress the importance of a better representation of polar regions in macroecological research, especially in light of the disproportionately rapid climatic change occurring at high latitudes (Previdi et al., 2021).

Another interesting similarity between elevational and latitudinal gradients is the stronger decrease in the intensity of trophic interactions for the gradients located entirely at higher elevations and higher latitudes than for the gradients located at lower elevations and lower latitudes respectively. This similarity may be explained by a stronger decrease in temperature per unit distance at both high elevations (Guo et al., 2016) and high latitudes (Wang & Dillon, 2014).

Despite an overall decrease in the intensity of trophic interactions with the increase in both latitude and elevation, these gradients differ greatly in the relative strength of the changes observed in individual interactions. These dissimilarities likely result from different changes in the abiotic environment along these two types of geographical gradients and from different sensitivities of herbivores, carnivores and parasites to similar changes in abiotic and biotic factors.

## CONCLUSION

The novelty of our study lies in (1) statistical testing for generality of patterns uncovered by case studies and narrative reviews; (2) discovery of previously unknown sources of variation in elevational patterns in biotic interactions, for example dramatic difference between trophic levels and geographic variation in strength of elevational decrease and (3) discovery of important similarities and dissimilarities between latitudinal and elevational changes in the intensity of trophic interactions. Our analysis of elevational changes in trophic interactions revealed considerable limitations for the prediction of a general decrease in their intensity with increased elevation. While carnivory and parasitism consistently decline from low to high elevation, for herbivory this prediction is unequivocally fulfilled only for ectothermic, openly living folivores at their background population densities in elevational gradients that cross tree lines in mountains located outside the polar zone. The differences between the outcomes of the two meta‐analyses conducted using similar methodologies (Zvereva & Kozlov, 2021; and the current study) suggest that elevational and latitudinal patterns in trophic interactions are to the great extent shaped by different mechanisms. Therefore, the scope of the LBIH could not be extended to incorporate elevational gradients.

## AUTHOR CONTRIBUTIONS

ELZ and MVK formulated goals and designed methodology, ELZ extracted data for meta‐analysis, conducted meta‐analysis and wrote the first draft of the manuscript, MVK participated in the writing of later drafts.

## Data Availability

The datasets used for the analyses are archived in Dryad Digital Repository (https://doi.org/10.5061/dryad.q573n5tms).
